# Influence of Environmental Conditions and Genetic Background of *Arabica* Coffee (*C. arabica L*) on Leaf Rust (*Hemileia vastatrix*) Pathogenesis

**DOI:** 10.3389/fpls.2017.02025

**Published:** 2017-11-28

**Authors:** Lucile Toniutti, Jean-Christophe Breitler, Hervé Etienne, Claudine Campa, Sylvie Doulbeau, Laurent Urban, Charles Lambot, Juan-Carlos H. Pinilla, Benoît Bertrand

**Affiliations:** ^1^Centre de Coopération Internationale en Recherche Agronomique pour le Développement, UMR IPME, Montpellier, France; ^2^Nestlé R&D Tours, Tours, France; ^3^Institut de Recherche pour le Développement, UMR IPME, Montpellier, France; ^4^UMR QualiSud, Université d’Avignon et des Pays du Vaucluse, Avignon, France

**Keywords:** *Coffea arabica*, coffee leaf rust, biotic–abiotic interaction, chlorophyll *a* fluorescence, hybrid vigor

## Abstract

Global warming is a major threat to agriculture worldwide. Between 2008 and 2013, some coffee producing countries in South and Central America suffered from severe epidemics of coffee leaf rust (CLR), resulting in high economic losses with social implications for coffee growers. The climatic events not only favored the development of the pathogen but also affected the physiological status of the coffee plant. The main objectives of the study were to evaluate how the physiological status of the coffee plant modified by different environmental conditions impact on the pathogenesis of CLR and to identify indicators of the physiological status able to predict rust incidence. Three rust susceptible genotypes (one inbred line and two hybrids) were grown in controlled conditions with a combination of thermal regime (TR), nitrogen and light intensity close to the field situation before being inoculated with the rust fungus *Hemileia vastatrix*. It has been demonstrated that a TR of 27-22°C resulted in 2000 times higher sporulation than with a TR of 23–18°C. It has been also shown that high light intensity combined with low nitrogen fertilization modified the CLR pathogenesis resulting in huge sporulation. CLR sporulation was significantly lower in the F1 hybrids than in the inbred line. The hybrid vigor may have reduced disease incidence. Among the many parameters studied, parameters related to photosystem II and photosynthetic electron transport chain components appeared as indicators of the physiological status of the coffee plant able to predict rust sporulation intensity. Taken together, these results show that CLR sporulation not only depends on the TR but also on the physiological status of the coffee plant, which itself depends on agronomic conditions. Our work suggests that vigorous varieties combined with a shaded system and appropriate nitrogen fertilization should be part of an agro-ecological approach to disease control.

## Introduction

Coffee is a product of mass consumption; with an estimated 2.4 billion cups consumed per day worldwide and with an average of 2.4% annual growth over the last 10 years, coffee is one of the world’s favorite beverages (International Coffee Organization, 2016). The annual turnover is approximately 30 billion Euros. Although coffee is not a food crop, it represents a major foreign exchange for earner in many developing countries (Ponte, 2002). The genus *Coffea* comprises approximately 124 species (Davis, 2011) but only two are cultivated at world scale: *Coffea* *arabica* and *Coffea canephora* (Wintgens, 2012). In 2015, *C. arabica* accounted for 58% of coffee production worldwide (International Coffee Organization, 2016). Whereas *C. arabica* originated in Ethiopia, today, the main cultivation areas are in South and Central America, which together account for more than 80% of world coffee production (Bertrand et al., 2012). The optimum mean annual temperature for Arabica coffee ranges from 18°C to 21°C (DaMatta and Cochicho Ramalho, 2006). Arabica coffee originates in humid forest, i.e., growing under shade conditions. However, the Arabica coffee tree has enough plasticity to be cultivated in both full sun and shade, although large quantities of external inputs are required under full sun (Matos et al., 2009).

Coffee leaf rust (CLR), the main fungal disease affecting coffee production worldwide, is caused by the biotrophic basidiomycete *Hemileia vastatrix* Berkeley and Broome (Basidiomycota, Pucciniales) and occurs in almost all producing countries (Silva et al., 2006). It is considered to be the most devastating disease for this culture. The pathogen affects living leaves and causes chlorotic lesions on the underside of the leaves. This reduces the photosynthetic area and in severe attacks, defoliation can occur leading to die-back of branches with heavy losses for farmers. The optimum temperature for germination is between 22°C and 24°C (de Jong et al., 1987; Rozo et al., 2012). Within the *Coffea* genus, *C. arabica* is subject to the most severe attacks with up to 30% losses if the disease is not controlled (Rozo et al., 2012).

From 2008 to 2013, more intense coffee rust epidemics than those previously observed occurred in Mesoamerica, from Colombia to Mexico, including Peru, Ecuador and some Caribbean countries (Avelino et al., 2015). These outbreaks were the worst since the disease first appeared in Central America in 1976. In Colombia, the incidence of the disease in the field increased from less than 5% before 2008 to more than 40% (Rozo et al., 2012) and was responsible for average reductions of 31 and 16% during the epidemic years compared with 2007 in Colombia (Cristancho et al., 2012) and Central America respectively. Because the majority of coffee is mainly produced by smallholders managing less than 10 ha of coffee (Jha et al., 2012), rust epidemics in Central America have had indirect impacts on food security. Several hypotheses have been proposed to explain the outbreak, including the emergence of a new virulence race of the pathogen and changes in plantation management. Indeed, fertilizer use declined due to the dramatic rise in prices during the 2008 global financial crisis, thus reducing the vigor of the coffee plants. Meteorological anomalies caused by the ongoing climate change are considered to be one of the main factors contributing to the emergence of the rust epidemic in 2012–2013, and to affect both the pathogen and the physiological status of the coffee tree (Rozo et al., 2012; Avelino et al., 2015). However, in a study focused only on pathogen response, Bebber et al. (2016) found that climate change had no effect on germination and appressorium formation and consequently did not significantly favor leaf infection.

For many years, breeding for CLR resistance was based on highly specific complete resistance derived from a major introgressed gene from the Hybrid of Timor (Herrera et al., 2008). However, in some countries, host resistance to CLR acquired from introgressive breeding with Hybrid of Timor clones as donor parent has turned out not to be durable (Van der Vossen et al., 2015). Developing strategies to improve the durability of rust resistance in Arabica cultivars based on genetic mechanisms combining both partial and complete resistance genes along with appropriate cultural practices, may be the best way to control this disease.

Although plant resistance is genetically controlled, the environment and particularly cultural practices can affect plant tolerance or resistance to pathogens by affecting plant physiology, the pathogen or both (Dordas, 2008). A survey conducted in 2014 in Nicaragua on coffee plantation showed that properly fertilizer and fungicide application limit rust incidence even during strong rust epidemic (Avelino et al., 2015). Shade has been proposed as a way of reducing *H. vastatrix* urediniospore dispersal. In a recent study, Boudrot et al. (2016) said that shade had opposite effects on urediniospore dispersion. Shading practices may help to supress or inversely may enhance the aerial dispersal of this pathogen depending on the rainfall regime. Pioneer studies on rust showed that the vegetative vigor of cereals reduced disease incidence (Raines, 1922). A preliminary study on coffee demonstrated that high productivity is positively correlated with the incidence of rust. In the Java variety, coffee trees from which 100% of the fruits had been removed subsequently had only 3% of infected leaves versus 60% in coffee trees with a yield of two tons per hectare (Bouharmont, 1995). A field survey strengthened these results by showing that a heavy fruit load enhanced rust infection (Avelino et al., 2006). It is generally known that good agronomic practices help control epidemics. These studies suggest that the leaf to fruit ratio could be a major determining factor of the reaction of the plant to the infection. Indeed, in coffee plants, fruits are typical sink-organs that influence the source sink balance as well as the physiological status of the tree (Vaast et al., 2006).

In some species, hybrid vigor has been shown to enhance their immune response (LeBoldus et al., 2013). Groszmann et al. (2015) showed that in Arabidopsis, hybrid vigor led to a different growth-defense balance with a higher growth rate and reduced basal defense gene activity, which, however, did not compromise their ability to set up a defense response comparable to that of the parents. Preliminary observations in coffee (by the authors of the present study) suggest that susceptible F1 hybrids are less severely attacked by CLR than pure lines. The use of F1 coffee hybrids could thus be a viable short-term alternative to seeking durable resistance against CLR.

The main objectives of the present study were thus (i) to evaluate how the physiological status of the coffee plant modified by different environmental conditions influences the pathogenesis of *H. vastatrix* and (ii) to identify indicators of the physiological status able to predict rust incidence.

We compared three susceptible genotypes (the inbred Caturra line and two F1 hybrids) using a combination of multiple stresses close to real field conditions (nitrogen fertilization, shade) before inoculating the coffee plant with the rust fungus *H. vastarix*. Before investigating the impact of nitrogen and shade on rust incidence in three genotypes, the inbred line Caturra has been studied under two thermal regimes (TRs) to identify the most suitable TR to analyze the impact of the physiological status of the coffee plant (*Coffea arabica*) on the incidence of rust.

## Materials and Methods

### Plant Material and Cultivation in a Phytotron

Three rust susceptible genotypes were studied: one inbred line *Coffea arabica* var Caturra and two Arabica hybrids: GPFA 109 and GPFA 124, F1 hybrids from the former. The Caturra seeds came from the La Cumplida research center (Matagalpa, Nicaragua). The two intraspecific Arabica hybrids GPFA109 and GPFA124 were vegetatively propagated by somatic embryogenesis at the Nestlé R&D laboratory (Tours, France). Both hybrids were selected for high cup quality, high productivity and excellent growth behavior. They are not expected to carry any specific genetic resistance to prevalent rust races. The plants were cultivated in a phytotron (65–75% humidity, 12 h day/12 h night) at CIRAD (Montpellier, France) in 3 L pots containing a 50:50 GO M2 (Jiffygroup) and N°9 (Neuhaus) potting soil mixture. Water was supplied every day and 39 mg of MS/2 medium (Murashige and Skoog, 1962) and 3 mg of KCl were applied to each coffee plant once a week for 11 weeks.

Eight Thermal regime (2) × Nitrogen fertilization (2) × Light intensity (2) treatments were compared. For each condition, four plants were studied for rust infection and fluorescence measurements, and three plants for chemical analysis. The two TR, i.e., day/night temperatures tested were: TR (27–22°C) and TR (23–18°C). Each coffee plant in the low nitrogen fertilization group was fertilized with a solution containing 35 mg of NH_4_NO_3_ once a week for 11 weeks, i.e., a total of 134 mg of nitrogen, whereas each coffee plant in the high nitrogen fertilization group was fertilized with a solution containing 69 mg of NH_4_NO_3_ per week, i.e., a total of 266 mg of nitrogen. The two light intensity levels tested were a photosynthetically active radiation (PAR) of 300 μmol⋅m^-2^s^-1^ i.e., low light intensity, and a PAR of 1000 μmol⋅m^-2^s^-1^ i.e., high light intensity, mimicking the light intensity perceived in an agroforestry system, and in a full sun system respectively.

### Rust Inoculation

The inoculum was composed of a mixture of urediniospores of *H. vastatrix* collected from Caturra, wild Ethiopian and Castillo coffee plants at the Cenicafé “Naranjal” experimental station located in the central coffee cultivation region in Colombia. This population of urediniospores mimicked the field CLR races currently found in the field.

The inoculation suspension was prepared by adding 0.5 mg of urediniospores per ml of sterile distilled water. The suspension was shaken for 30 s under sonication. To test the viability of the spores, 20 drops (5 μl each) of the inoculation suspension were cultured in 1% agar-water medium in Petri dishes. The dishes were kept in the dark at 20°C for 12 h. After the spores were stained with lactophenol, the germination rate of at least 500 spores was evaluated. Observations were made with a 40x binocular magnifier. An inoculum was considered suitable for rust infection when the germination rate was over 60%.

The leaves of 6-month-old plantlets (around 35 cm in height) were inoculated by spraying a suspension of urediniospores over the lower surface of each leaf of each tree. After inoculation, the plants were kept for 48 h in the dark at 23°C and 100% relative humidity.

### Macroscopic Monitoring of the Infection

The infection was monitored macroscopically in quadruplicate using a completely random experimental design. To characterize the infection, two indicators of rust incidence were measured on each coffee plant:

#### The Percentage of Infected Leaves during the Time Course of the Infection

The percentage of leaves with at least one sporulating lesion was determined 21, 24, 37, 43 days post-inoculation (dpi) for TR (27–22°C) and 24, 30, 35, 43 days post-inoculation (dpi) for TR (23–18°C).

#### Quantity of Spores per Infected Leaf Area Produced by *H. vastatrix*

At 43 dpi, the rust was harvested from each leaf of each coffee plant and weighted per tree. Leaf area was measured on leaves with at least one pustule. The quantity of spores per infected leaf area represents the ratio of the weight of the rust to the cumulated infected leaf area per tree.

### Chemical Analyses

Samples for chemical analysis were collected 1 day before inoculation at Zeitgeber time = 10 h (ZT10), on plants cultivated in the same conditions as the inoculated plants. Leaves were immediately frozen in liquid nitrogen, and kept at -80°C until freeze-drying. Analyses were performed in triplicate on three independent plants and three different extractions, using a completely random experimental design.

#### Determination of Mineral Elements

Mineral elements were determined by the CIRAD US-Analyses laboratory. Nitrogen was determined using the Dumas method. Roughly 150 mg of dry powder was weighed precisely in tin foil and analyzed using Leco’s Nitrogen Determinator (model FP-528, Leco Corp., St. Joseph, MI, United States). The nitrogen content (in milligrams per gram) was determined using calibration curves set up using commercial standards of EDTA and orchard leaves. The other elements (P, K, Ca, Mg, Fe, Cu, Zn, and B) were determined by inductively coupled argon plasma atomic emission spectrometry (ICP-OES) after dry mineralization. Samples were analyzed by ICP-OES using an Agilent 720-ES equipped with a CCD detector. NO_3_^-^ and NH_4_^+^ were extracted from 500 mg of dry powder and quantified with a continuous-flow colorimeter. All elements were determined in triplicate and are expressed as a percentage of dry weight (% DW) or parts per million (ppm).

#### Sugar Metabolism

Sugars were extracted from 20 mg samples of freeze-dried powder and measured by high performance anion exchange chromatography coupled with pulsed amperometric detection (Dionex Chromatography Co., Sunnyvale, CA, United States) as described in Dussert et al. (2006).

The starch content of 30 mg of freeze-dried powder was determined using the total starch kit GOPOD (D-glucose, K-Gluc, Megazyme International, Ireland). After elimination of soluble sugars and of the soluble products of starch degradation, the residue was successively hydrolysed into glucose units with α-amylase and amyloglucosidase. The resulting D-glucose was then degraded with glucose oxidase and the resulting hydrogen peroxide quantified by spectrophotometry at 510 nm after a last enzymatic reaction. Results are expressed as % DW.

#### Phenolic Extraction and Quantification

The lyophilised plant material was ground in a ball mill (TissueLyser II, Qiagen) and extracted at 4°C for 3 h under stirring (225 rpm, Rotamax 120, Heidolph) using 25 mg of plant material in 6 mL of MeOH/H2O (80:20, v/v). After centrifugation for 8 min at 3500 rpm, the methanol extract was collected and filtered (Millipore, 0.25 μm porosity) before analysis. Each sample was characterized by its mean concentration of purine alkaloid (caffeine), hydroxycinnamic acid esters (caffeoylquinic acids, dicaffeoylquinic acids, one feruloylquinic acid isomer), xanthone (mangiferin) and flavonoids [(+)-catechin, (-)-epicatechin], expressed as % DW. Quantification was carried out on 10 μL of extract using a HPLC system (Shimadzu LC 20, Japan) equipped with a photodiode array detector consisting of an eclipse XDB C18 (3.5 μm) column (100 mm × 4.6 mm, Agilent). The elution system (0.6 mL min^-1^) was comprised of two filtered (0.2 μm pore size filter), sonicated and degassed solvents, solvent A (water/acetic acid, 98:2, v/v) and solvent B (H2O/MeOH/acetic acid, 5:90:5 v/v/v). The linear gradient was 0 min, 15% solvent B; 0–4 min, 25%; 4–8 min, 32%; 8–10 min, 35%; 10–14 min, 58%; 14–16 min, 62%; 16–18 min, 64%; 18–21 min, 80%; 21–24 min, 15%; 24–26 min, isocratic.

The calibration curve was plotted using three replicate points of standard solutions of caffeine, mangiferin, 5-CQA, purchased from Sigma–Aldrich Chimie (St Quentin Fallavier, France), glucosylated kaempferols and quercetin, rutin, (+)-catechin, (-)-epicatechin and epigallocatechin from Extrasynthese (Lyon, France) and 3,5-*o*-dicaffeoylquinic acid (3,5-diCQA) from Biopurify Phytochemicals (Chengdu, China) at 25, 50, 75, and 100 μg mL^-1^. Identification was performed by comparing spectra and retention times at 280, 320, and 360 nm. Quantification of caffeoylquinic acids (3-, 4-, and 5-CQA), feruloylquinic acids (one FQA isomer) and dicaffeoylquinic acids (3,4-, 3,5- and 4,5-diCQA) was performed at 320 nm, caffeine and catechin derivatives at 280 nm, and mangiferin, kaempferol and quercetin derivatives at 360 nm, by comparison with their respective standards.

### Phenotyping

One day before inoculation, plant height, and the fresh weight and dry weight of the roots, shoots and the entire plant, stomatal density and specific leaf weight were measured for each condition on 4 plants per genotype.

In order to measure stomatal density, small leaf pieces (1 cm^2^), were observed and photographed with a DM600 LAICA microscope under the 20X magnification (1 pixel = 0.3659 μm). Four fields of 0.56 mm^2^ per piece, i.e., 2.84% of piece surface were studied.

### Chlorophyll *a* Fluorescence Measurements

Chlorophyll *a* fluorescence measurements were conducted between ZT9 and ZT11 with a Handy PEA chlorophyll fluorimeter (Handy-Plant Efficiency Analyser, Hansatech Instruments, Norfolk, United Kingdom) on mature leaves. Measurements were performed in quadruplicate. Four fluorescence measurements per plant were performed. Leaves were dark-adapted for 20 min prior to measurement. When leaves kept in the dark are illuminated, chlorophyll *a* fluorescence intensity shows characteristic changes called fluorescence transient (Stirbet and Govindjee, 2011). Chlorophyll *a* fluorescence transients were induced by 1 s illumination with an array of six light-emitting diodes providing a maximum light intensity of 3000 PAR. The fast fluorescence kinetics (from F_0_ to F_M_, where F_0_ and F_M_ are, respectively, the minimum and maximum measured chlorophyll fluorescence of PSII in the dark-adapted state) was recorded from 10 μs to 1 s. Dark-adaptation allowed the PSII electron acceptor pool to be gradually re-oxidized to a point where all PSII reaction centers are capable of photochemistry. Fluorescent transients were analyzed using the JIP test developed by Strasser and Strasser (1995). The JIP test evaluates the balance between total energy inflows and outflows and provides the probable distribution of light energy absorption (ABS) between the events: trapping (TR), electron transport (ET) and dissipation (DI) (Kalaji et al., 2016). Some parameters were analyzed in more detail: (1) The average absorbed photon flux per PSII reaction center (J^abs^/RC), which expresses the apparent antenna size of an active PSII; (2) The maximum quantum yield of photosystem II (F_V_/F_M_ = TR/ABS), which expresses the trapping flux/ absorption flux. This describes the performance of the light reaction; (3) The pool size of electron carriers per RC of PSII [Sm = Area/(F_M_-F_o_)], which is proportional to the number of electrons passing through the electron transport chain; (4) The quantum yield of the electron transport flux up to the PSI electron acceptors (J_0_^RE1^/J^abs^), which expresses the rate of electron transport from QB to PSI acceptors (Stirbet and Govindjee, 2011).

### Statistical Analyses

All statistical analyses were performed using R 3.2.4 software.

For each treatment, a Student’s test was performed to test the effect of the TR on the percentage of infected leaves and on the quantity of spores per infected leaf area at 43 dpi (stats package). Tests for normality and equality of variance (Shapiro test and Levene test, car package) were performed. Alternatively, the non-parametric Mann–Whitney–Wilcoxon test was performed (stats package).

One-way ANOVA of the two indicators of rust incidence and on all metabolic and phenotyping variables was performed to test the effect of the different treatments and genotypes. Prior to each ANOVA, tests for normality and equality of variance were performed (Shapiro test and Levene test, car package) and significant effects were analyzed by multiple comparisons of means (Tukey honest significant differences test, stats package). Data that did not conform to conditions of normality and homoscedasticity were log transformed. When the conditions of normality were not fulfilled despite the logarithm transformation, a non-parametric Kruskal–Wallis test was performed (pgirmess package).

Logarithmic regression between parameters derived from Chlorophyll *a* fluorescence induction curve and the quantity of spores per infected leaf area at 43 dpi were fitted using non-linear least square for estimating parameters (stats package). The equation was aa^∗^log10(X) + bb. Starting values were initiated to a = 0.5 and b = 1. Only points corresponding to high light intensity were fitted.

## Results and Discussion

**FIGURE 1:**
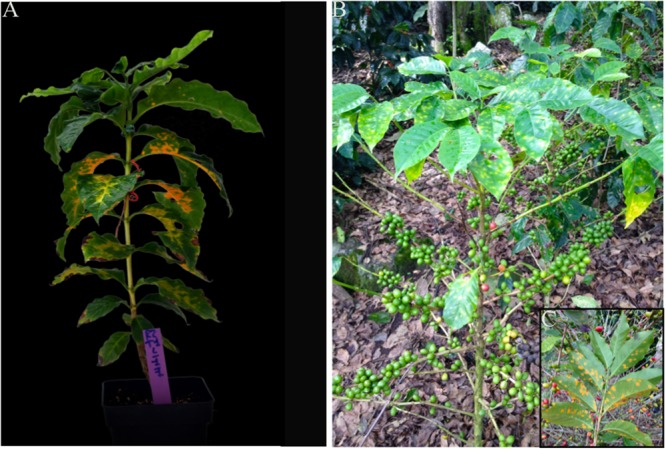
Arabica coffee plants infected by *H. vastatrix*. Symptoms in the controlled conditions of this study **(A)** were quite similar to those observed in field conditions **(B,C)**.

The level of the rust infection of three genotypes have been compared (one inbred line and two F1 hybrids) cultivated under different combinations of agronomic conditions close to those found in real field conditions (nitrogen fertilization, light intensity) (**Figure 1**). Two TRs were tested, the first corresponding to the most suitable TR for the production of Arabica coffee and the second corresponding to the probable future TR in Arabica cultivation area under continuing global warming (Bunn et al., 2015).

### Effect of Thermal Regime on CLR Incidence

**FIGURE 2:**
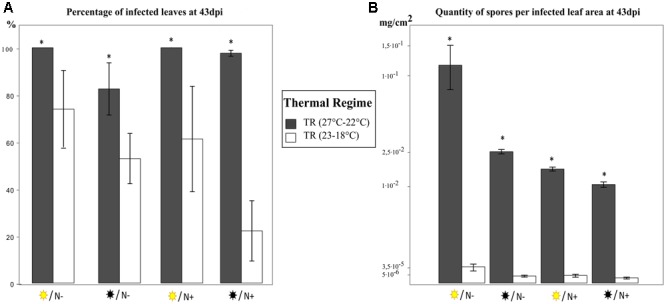
Comparison of two thermal regimes in the inbred line Caturra with different combinations of light intensity and nitrogen fertilization 43 days post *H. vastatrix* inoculation. **(A)** Percentage of infected leaves. **(B)** Quantity of spores per infected leaf area. The quantity of spores per infected leaf area is plotted on a cube root scale in order to present the two thermal regimes on the same plot despite the marked differences between the values. Four treatments were studied 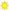/N- 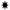/N- 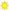/N+ 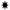/N+ corresponding to respectively high light intensity (1000 PAR)/low nitrogen fertilization, low light intensity (300 PAR)/low nitrogen fertilization, high light intensity (1000 PAR)/high nitrogen fertilization, low light intensity (300 PAR)/high nitrogen fertilization. The thermal regime 27°C–22°C is in black and the 23°C–18°C regime is in white. The data are means ± SD (*n* = 4). Within each treatment, means from thermal regimes followed by an asterisk are significantly different according to Student’s test (*P* < 0.05).

Caturra and closely related varieties (Mundo novo, Catuai, Typica) currently account for 80% of coffee orchards worldwide. Consequently, we will first present the results of the two TRs focusing on Caturra in order to highlight the strong effect of TR on rust incidence before considering genotypes and cultural practices effects (**Figure 2**).

**FIGURE 3:**
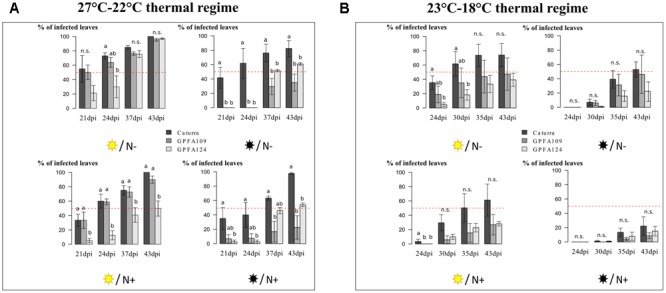
Variations in the percentage of leaves infected by *H. vastatrix* during the time course of the infection under different combinations of light intensity and nitrogen fertilization in three different genotypes. **(A)** The 27°C–22°C thermal regime. **(B)** The 23°C–18°C thermal regime. For each thermal regime, four treatments were studied 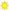/N- 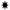/N- 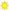/N+ 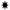/N+ corresponding respectively to high light intensity (1000 PAR)/low nitrogen fertilization, low light intensity (300 PAR)/low nitrogen fertilization, high light intensity (1000 PAR)/ high nitrogen fertilization, low light intensity (300 PAR)/high nitrogen fertilization. The inbred line Caturra and the two hybrids GPFA109 and GPFA124 are respectively in black, dark gray and light gray. The red dotted line represents 50% of infected leaves. The data are means of the percentage of infected leaves ± SD (*n* = 4). Different letters denote significant differences between the genotypes within each treatment according to Tukey’s test (*P* < 0.05). ns, non-significant differences.

The 27–22°C TR was the most favorable TR for rust (**Figure 2A**). Under TR (27–22°C), regardless of the light intensity and nitrogen fertilization: at least 80% of Caturra leaves were infected at 43 dpi. By contrast, under TR (23–18°C) the mean percentage of infected leaves never reached 80%. The TR affected the quantity of spores produced even more intensely (**Figure 2B**). The quantity of spores per infected leaf area was 10,000 times higher under TR (27–22°C) than under TR (23–18°C) and a cube root scale was needed to plot the two TRs on the same graph. Consequently, the best indicator to study the influence of the different treatments on rust incidence appears to be the quantity of spores per infected leaf area not only because it discriminated the different treatments better but also because it will have the most impact for the farmers (**Figure 2**). In Caturra, the latent period (defined as the period from inoculation until the formation of pustules) was longer under TR (23–18°C) than under TR (27–22°C) (**Figure 3**). Under TR (23–18°C), the percentage of infected leaves reached 50% on average 11 days later than under TR (27–22°C) resulting in slower infection.

The role of weather particularly of temperature in the likelihood of disease outbreak is well known to farmers. Both pioneer and recent studies showed that the germination of *H. vastatrix* urediniospores and subsequent penetration into the leaf via stomata is highly dependent on surface wetness and temperature (Nutman and Roberts, 1963; Bebber et al., 2016). The optimum temperature for germination reported in the historical and recent studies was not the same, suggesting that *H. vastatrix* adapted to warmer temperatures. de Jong et al. (1987) found the optimum temperature for germination to be 22°C, whereas Rozo et al. (2012) reported 24°C to be the optimum temperature. In our experiment, all the coffee plants were kept at a constant temperature of 23°C for 48 h following inoculation before being subjected to the two TRs. The effect of the temperature observed in our study showed that the temperature not only affects germination but also affected later stages of fungal development (i.e., colonization of host tissue). Kushalappa (1989) reported that an increase in temperature was associated with a decrease in the length of the latent period of *H. vastatrix*, resulting in more rapid infection. Temperature has also been shown to have a notable effect on the rapidity of infection by other fungal pathogens. Magarey et al. (2005) adapted a temperature response function to create a model for predicting infection periods by fungal foliar pathogens and validated it with a large experimental data set. In peppermint rust, Edwards et al. (1998) reported that the latent period of infection was five times shorter at 22°C than at 5°C. Even if the effect of the environment on the sporulation process of *H. vastatrix* is well known (Kushalappa, 1989), the link between the quantity of spores produced and the temperature has not been yet investigated. In peppermint, a significant effect of temperature on *Puccinia menthae* sporulation has been reported. Daily spore production increased more than three times with an increase in temperature from 5°C to 20°C and was inhibited at 27°C (Edwards et al., 1998).

The low rust incidence under TR (23–18°C) compared to under TR (27–22°C) was mainly due to limitation of *H. vastatrix* sporulation by temperature. TR (27–22°C) was the TR under which cultural practices with nitrogen fertilization and agroforestry versus full sun field conditions had the largest impact on rust incidence. Hence, the presentation of the results will be focused on this TR.

### Effect of Light Intensity and Nitrogen Fertilization on CLR Incidence in the Inbred Line Caturra

**FIGURE 4:**
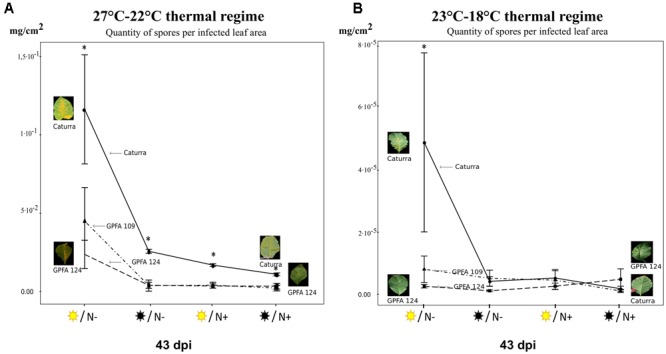
Quantity of spores per infected leaf area (also named ‘sporulation’) produced by *H. vastatrix* under different combinations of light intensity and nitrogen fertilization in three different genotypes. **(A)** The 27°C–22°C thermal regime. **(B)** The 23°C–18°C thermal regime. NB: The scale of the two graphs is different. For each thermal regime, four treatments were studied 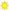/N- 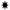/N- 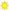/N+ 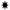/N+ corresponding respectively to high light intensity (1000 PAR)/low nitrogen fertilization, low light intensity (300 PAR)/low nitrogen fertilization, high light intensity (1000 PAR)/high nitrogen fertilization, low light intensity (300 PAR)/high nitrogen fertilization. Circles represent the inbred line Caturra, triangles the hybrid GPFA109 and squares GPFA124. The data are means of the spores weight harvested on the coffee plant 43 days post inoculation per infected leaf area ±SD (*n* = 4). Within each treatment, means for a genotype followed by an asterisk are significantly different according to Tukey’s test (*P* < 0.05).

Low light intensity and high nitrogen fertilization slowed down infection and sporulation in the inbred line Caturra (**Figures 3, 4**). Under TR (27–22°C), low light intensity delayed infection in Caturra (**Figure 3A**). The mean percentage of infected leaves reached 66% at 24 dpi under high light intensity whereas only 50% of the leaves were infected under low light intensity on average over the two nitrogen fertilization levels. In Caturra, infection was slowed down by high nitrogen input (**Figure 3A**). At 24 dpi, the mean percentage of infected leaves was 50% with high nitrogen inputs versus 67% with low nitrogen inputs on average over the two light intensity levels. The pattern was the same under TR (23–18°C), i.e., infection was slower under the lowest light intensity and faster under a low nitrogen fertilization even when sporulation was very weak (**Figure 3B**). The quantity of sporulation was affected in the same way by nitrogen and light intensity, suggesting that lower rust penetration and/or colonization of host tissue limited sporulation (**Figures 3, 4**). However it was not the only factor that limited sporulation quantity. Under TR (27–22°C) and high light intensity, minor differences in the time course of infection between the different levels of nitrogen fertilization led to a marked increase in the quantity of sporulation with low nitrogen fertilization suggesting a higher quantity of spores per lesion (**Figures 3A, 4A**). Whatever the TR, high light intensity combined with low nitrogen fertilization led to a significantly higher quantity of spores at 43 dpi compared to with the other treatments (**Figure 4**). These results suggest that agroforestry practices associated with sufficient nitrogen fertilization could limit rust infection in the susceptible inbred line Caturra.

Avelino et al. (2006) monitored the development of rust epidemics in 73 plots in Honduras focussing on coffee plant characteristics, crop management and the environment. These authors showed that yield and fertilization had the most effect on coffee rust. No fertilization in the plots with good fruit yields was in most cases associated with high rust values. Nitrogen is an essential macronutrient for optimum coffee growth and is an essential component of fertilization in coffee culture. Hence, our study focussed on nitrogen fertilization. The effect of N on disease development has been shown to vary in different studies, suggesting that the effect of N-supply on susceptibility is pathogen-specific (Dordas, 2008). Ramalho et al. (1997) showed that N starvation associated with high light exposure caused a marked reduction in photosynthetic capacity of coffee plants, and concluded that nitrogen availability is a key factor in acclimation to high light intensity. This less favorable physiological status could explain the high incidence of rust in our study when high light intensity was combined with low nitrogen fertilization. Avelino et al. (2006) found that shade enhanced rust infection, which seems to contradict our results. However, in the study in Honduras, shade mainly affected the rust germination stage by providing a humid environment, low intensity light and buffer temperatures. In our study, all the coffee plants were kept in the same conditions during germination to avoid any effect of environmental conditions on germination. Germination rates have been measured *in vitro* and shown to be similar under all treatments. In our study, the lower percentage of infected leaves under low light intensity suggests a lower penetration rate or a lower host tissue colonization of *H. vastatrix* under shade.

### Effect of Genotype (Hybrids versus Inbred Line) on CLR Incidence

In the hybrids, rust infection was delayed and resulted in less sporulation (**Figures 3, 4**). Whatever the TR and the combination of light intensity and nitrogen fertilization, rust infection was slower in the hybrids than in the inbred line (**Figure 3**). The time course of infection was very similar in the two hybrids under all treatments except one. In the case of TR (27–22°C), under high light intensity, the time course of the infection in GPFA 109 hybrid was very similar to that in Caturra, whereas infection was much slower under low light intensity compared to in the inbred line (**Figure 3A**). The difference in the time course between the inbred line Caturra and the hybrids was maximized under TR (27–22°C) with low light intensity and low nitrogen fertilization (**Figure 3A**). The latent period lasted approximately 21 days for Caturra versus 37 days for the hybrids suggesting a reduced penetration or colonization of host tissue in the hybrids. The superiority of the hybrids in the shade was expressed by the less intense rust attack compared to Caturra. It is noteworthy that F1 hybrids were affected by nitrogen and light intensity in a similar way to Caturra. Hence, infection was slower under low light intensity and faster under low nitrogen fertilization (**Figure 3**).

Under TR (27–22°C), the quantity of spores produced per infected leaf area was always significantly lower in the hybrids than in the inbred line Caturra (**Figure 4A**). Regardless of the combination of light intensity and nitrogen fertilization, the quantity of spores was similar in the two hybrids. Under TR (23–18°C), the quantity of spores produced was significantly lower in the hybrids than in the inbred line only when the coffee plants were grown under high light intensity and low nitrogen fertilization (**Figure 4B**). The hybrids were more homeostatic in the quantity of spores than the inbred line Caturra (**Figure 4**).

Other authors have drawn contradictory conclusions about the better resistance of hybrids to disease. Groszmann et al. (2015) showed that the better growth of the Arabidopsis hybrids reduced basal defense gene activity but did not affect their defense ability against *Pseudomonas syringae* compared to that of their parents. Conversely, a study on willow leaf rust showed that the hybrids were more susceptible than their parents (Roche and Fritz, 1998).

Under the TR (27–22°C) TR, which will probably prevail in the Arabica cultivation area in a few years, the time course of the infection was delayed and sporulation was lower in the hybrids regardless of the combination of light intensity and nitrogen fertilization (**Figures 3, 4**). Under low light intensity, the time course of the infection was strongly delayed and sporulation was significantly lower in the hybrids. Under high light intensity, whereas the difference between the time courses of infestation was less pronounced, the quantity of rust sporulation was still lower, perhaps due to fewer spores per lesion in the hybrids. These results highlight the superiority of the hybrids with respect to rust infection in agroforestry systems, because both the penetration/colonization of host tissue and the intensity sporulation are limited.

### Study of the Link between Indicators of Coffee Plant Physiological Status and CLR Incidence

It has been demonstrated that genotype and cultural practices have an impact on CLR incidence. We hypothesized that those latter impacted CLR incidence by modifying physiological state of coffee plants. To investigate the link between indicators of the physiological status of coffee plants before inoculation and rust incidence, we considered TR (27–22°C) and, as an indicator of rust incidence, the quantity of spores per infected leaf area.

Plant height, fresh weight and dry weight of the roots, shoots and the entire plant, stomatal density and specific leaf weight were first measured for each condition before rust inoculation to visualize physiological state modification. Except for the specific leaf weight, no significant differences have been observed between the treatments for those phenotyping variables during the time of the experiment, Leaves are thicker under high light intensity (ANNOVA; *p*-value: 0.002). However, specific leaf weight was not correlated to CLR incidence. We hypothesized that because of the short duration of the experiment (3 months), different physiological state didn’t lead to phenotyping variation yet.

To check the link between physiological state of the coffee tree and CLR incidence, chlorophyll *a* fluorescence measurement and metabolic analysis have been performed.

**FIGURE 5:**
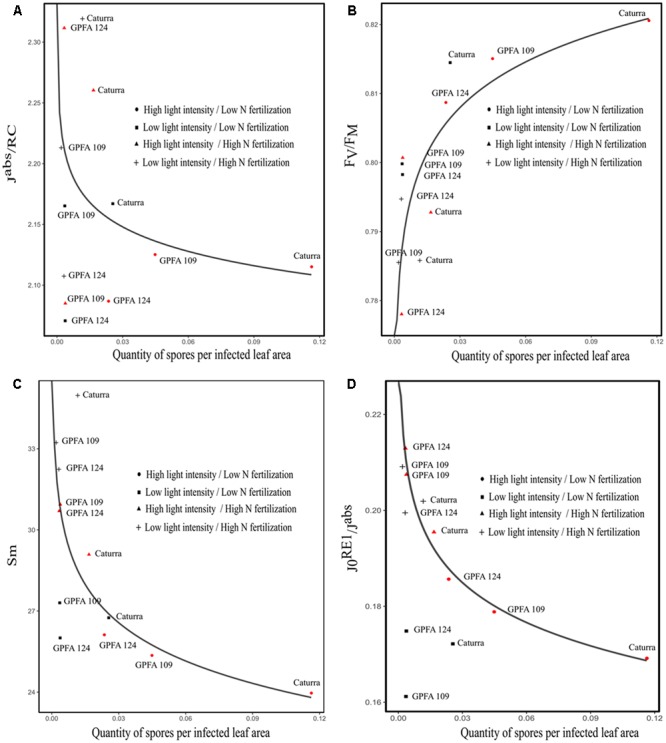
Correlation between parameters derived from Chlorophyll *a* fluorescence induction curve and the quantity of spores harvested on three *Coffea arabica* genotypes grown with different combinations of light intensity and nitrogen fertilization under TR (27–22°C). **(A)** The rate of photon absorption per PSII (J^abs^/RC). The points corresponding to light treatment (in red) were fitted by the equation *y* = -0.07^∗^log(x) +2.05; residual standard error: 0.091. **(B)** The maximum quantum yield of primary photochemistry (F_V_/F_M_). The points corresponding to light treatment (in red) were fitted by the equation *y* = 0.02^∗^log(x) +0.84; residual standard error: 0.009. **(C)** The normalized area proportional to the number of electron carriers per electron transport chain. The points corresponding to light treatment (in red) were fitted by the equation *y* = -4.7^∗^log(x) +19; residual standard error: 0.08. **(D)** The quantum yield of the electron transport flux until the PSI electron acceptors (J_0_^RE1^/J^abs^). The points corresponding to light treatment (in red) were fitted by the equation *y* = -0.03^∗^log(x) + 0.14; residual standard error: 0.003.

Chlorophyll *a* fluorescent transient of dark adapted leaves was performed at the end of the light period (from ZT9 to ZT11) before inoculation with *H. vastatrix*. Chlorophyll *a* fluorescence measurements provide indirect information about the physiological condition of plants. The JIP test has been used to analyze the polyphasic rise of the chlorophyll *a* fluorescence transient (OJIP) (Strasser and Strasser, 1995). It allows the evaluation of the physiological condition of photosystem II (PSII) and photosynthetic electron transport chain components. It has been investigated if the different combinations of light intensity and nitrogen fertilization led to different behavior in terms of photon absorption, photon trapping, electron transport and energy dissipation, and if these adaptive behaviors were correlated with rust sporulation. As previously shown, low light intensity strongly limited rust sporulation in the hybrids [see Effect of Genotype (Hybrids versus Inbred Line) on CLR Incidence]. Hence, to study the link between indicators of the physiological status of coffee plant before inoculation and the quantity of rust sporulation, the high light intensity treatment highlighted in red in **Figure 5** has been particularly studied.

Under the condition leading to higher rust incidence, the quantity of photon absorbed per PSII (J^abs^/RC) was low (**Figure 5A**). However, the trapping flux/ absorption flux (F_V_/F_M_) was high, meaning that the majority of the photons absorbed were trapped and very little energy flux was dissipated by processes other than trapping, such as heat dissipation (**Figure 5B**). Therefore, during exposure to full sunlight or when environmental conditions restrict plant growth, leaves are unable to utilize all the PAR absorbed and massive levels of excess excitation energy are encountered. This excess energy has the potential to be transferred into oxygen leading to ROS production and causing damage to the plant. One key mechanism to avoid photooxidative damage is the dissipation of excess energy as heat in the antenna pigment complexes of PSII before it reaches the PSII reaction centers (Demmig-Adams and Adams, 2006). When nitrogen deficiency is associated with high light intensity, excess energy is further increased by affecting biochemical reactions in the stroma, thereby reducing the activity of carboxylating enzymes, activities resulting in decreased CO_2_ fixation capacity (Correia et al., 2004). As a result, in our study, because coffee plants did not evacuate excess energy, ROS was probably produced, causing damage to the plant and explaining the high incidence of rust. Our results are consistent with those of DaMatta et al. (2007), who tested different source-sink balances in field-grown coffee trees. These authors found higher values of F_V_/F_M_ for leaves sampled in March on coffee trees under higher sink demand compared with leaves on coffee trees from which 50% of coffee fruits had been removed. In our study, some parameters pointed to impairment of the electron transport chain under the treatment which led to higher rust incidence (**Figures 5C,D**). Sm, assessing the number of electrons passing through the electron transport chain, was negatively correlated with the subsequent quantity of sporulation (**Figure 5C**). J_0_^RE1^/J^abs^ corresponding to the rate of electron transport from QB to PSI acceptors had low values. J_0_^RE1^/J^abs^ is assumed to provide insight into the cyclic electron flux which can limit photooxidative stress by favoring the ATP to NADPH output ratio (Ripoll et al., 2016). The negative correlation between J_0_^RE1^/J^abs^ and the quantity of rust sporulation reflects a decline in the cyclic flux, which favors rust sporulation by increasing photooxidative stress. (**Figure 5D**). Therefore, in Caturra, which was highly impacted by rust (high light intensity and low nitrogen fertilization), the hexose to sucrose ratio was significantly higher, reflecting strong inhibition of sucrose biosynthesis followed by the accumulation of hexoses (**Table 1**). Despite the high hexose: sucrose ratio, sucrose content was high, suggesting poor-sucrose export efficiency. Accumulation of soluble sugars negatively regulates photosynthesis gene expression including expression of Calvin cycle genes (Couee et al., 2006). There probably was an imbalance between the excitation energy and the quantity of NADPH produced and, on the other hand, the electron flux used by the Calvin cycle. This imbalance increased ROS production even more through poor recycling of NADP+. This result was corroborated by the high starch content (**Table 1**). Starch accumulation is a mechanism which prevents down regulation of photosynthesis by providing an outlet for photosynthetic end-products when exports fail to evacuate them effectively. Excessive starch could therefore be toxic (DaMatta et al., 2007). The accumulation of soluble sugars could be due to low nitrogen fertilization. In tomato, it has been demonstrated that N-deficient leaves increase the accumulation of starch and major soluble sugars and decrease accumulation of amino acids (Sung et al., 2015). In phenolic compounds, which are assumed to be defense compounds, the concentration of mangiferin and flavonoids before inoculation was higher in Caturra, which was highly impacted by rust (high light intensity and low nitrogen fertilization) suggesting a stress status but also pointing to the compounds’ inefficiency against rust (**Figure 4A** and **Table 1**). However, the concentration of di CQA, which is assumed to be a powerful antioxidant, was lower.

**Table 1:** Influence of two levels of nitrogen fertilization on indicators of the physiological state of three different *Coffea arabica* genotypes under TR (27–22°C) and high light intensity.

Parameters	Caturra		GPFA 109		GPFA 124	
	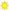 /N-	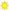 /N+	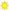 /N-	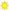 /N+	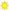 /N-	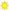 /N+
Hexoses/Sucrose	0.85 ± 0.09a	0.41 ± 0.2b	0.36 ± 0.2a	0.49 ± 0.1a	0.46 ± 0.1a	0.57 ± 0.08a
Sucrose (% DW)	3.9 ± 0.3a	4.08 ± 0.2a	2.3 ± 0.07a	2.57 ± 0.2a	3.4 ± 0.2a	3.19 ± 0.2a
Starch (%DW)	2.74 ± 0.8a	1.7 ± 0.4a	0.48 ± 0.04a	0.52 ± 0.008a	1.07 ± 0.2a	1.05 ± 0.3a
NH_4_ (PPM)	120 ± 6a	142.4 ± 7.7a	159.3 ± 1a	177.1 ± 1a	177 ± 6.1b	235.4 ± 4.2a
C /NH_4_	0.4 ± 0.019a	0.33 ± 0.017b	0.30 ± 0.02a	0.27 ± 0.026a	0.26 ± 0.009a	0.20 ± 0.0034b
Mangiferin (% DW)	0.43 ± 0.01a	0.29 ± 0.045b	0.65 ± 0.07a	0.66 ± 0.03a	0.42 ± 0.05a	0.42 ± 0.025a
Flavonoids (% DW)	0.99 ± 0.1a	0.71 ± 0.09b	1.06 ± 0.05a	1.17 ± 0.06a	0.84 ± 0.1a	0.88 ± 0.02a
DICQA (%DW)	0.46 ± 0.02b	0.54 ± 0.025a	0.80 ± 0.025a	0.79 ± 0.07a	0.69 ± 0.05a	0.7 ± 0.09a
CQA (%DW)	3.9 ± 0.07a	3.37 ± 0.17a	3.8 ± 0.06a	4 ± 0.19a	4.2 ± 0.28a	4.2 ± 0.021a

*Two treatments were studied: 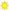/N- 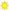/N+ corresponding to respectively high light intensity (1000 PAR)/low nitrogen fertilization and high light intensity (1000 PAR)/high nitrogen fertilization. The data are means ± SD (*n* = 3). Different letters denote significant differences between the two treatments according to Tukey’s test (*P* < 0.05).*

Whereas parameters related to physiological conditions of PSII and photosynthetic electron transport chain components seemed to explain the differences in the quantity of rust sporulation in Caturra and in the two hybrids under high light intensity, the behavior of the hybrids with respect to metabolic content appeared to be very different from that of Caturra (**Table 1**). GPFA 109 and GPFA 124 hybrids showed better homeostasis for all metabolites. However, the nitrogen utilization was more efficient in the hybrids than in Caturra, especially in GPFA 124. Regardless of the genotype, a higher leaf C to NH4 ratio was associated with higher quantity of spores per infected leaf area (Pearson correlation coefficient value: 0, 58; *p*-value: 0, 04). The C: N ratio has been widely studied and is considered to be a good indicator of the trade-off between growth and defense (Royer et al., 2013).

## Conclusion

The results obtained in controlled conditions demonstrate that high temperature favors pathogen development but also increases the impact of agricultural practices such as nitrogen fertilization and shade on the incidence of CLR in susceptible varieties. Higher temperature is one of the meteorological anomalies caused by ongoing climate change. Under the 27–22°C TR, shade and high nitrogen fertilization limited rust incidence probably by affecting both host tissue penetration/colonization and sporulation intensity. Moreover, vigorous varieties were less infected and more homeostatic than conventional varieties. The superiority of hybrids with respect to rust attack was particularly clear under the 27–22°C TR (the TR which will probably prevail in *Arabica* cultivation areas in a few years) and under low light intensity. Because of the different behavior of the hybrids compared to Caturra, metabolites could not be used as predictors for either Caturra or the hybrids. However, we recommend studying the C: NH4 ratio, which seems to predict the amount of sporulation in real conditions fairly well. Among the many parameters studied, those related to the physiological condition of photosystem II and photosynthetic electron transport chain components appeared to be useful indicators of the physiological status of the coffee plant and able to predict the capacity of the plant to resist the disease. To our knowledge, until now, Chlorophyll *a* fluorescence imaging has already been used to study physiological responses after pathogen attacks but not yet to assess the physiological state before infection. It could be a simple, non-destructive, inexpensive and rapid tool to detect the physiological state which will lead to high rust sporulation. Genetic resistance to rust based on major SH genes is becoming progressively less durable. Based on preliminary observations in the field (by the authors of the present study) suggesting that susceptible F1 hybrids are less severely attacked by CLR than pure lines, we confirm this result in controlled conditions. We showed that under shade and sufficient nitrogen fertilization, rust incidence is very low on F1 hybrid. Hence, our work suggests that vigorous varieties (mainly F1 coffee hybrids) combined with a shaded system and appropriate nitrogen fertilization could be a useful part of an agro-ecological approach to controlling this disease. We consider that the currents Arabica breeding programs mainly devoted to the selection of resistant varieties should also consider these results and reorient their objectives toward obtaining vigorous and resilient varieties.

## Author Contributions

LT, J-CB, HE, CL, J-CP, and BB designed the study. LT, J-CB, HE, and BB contributed to sample harvest and data analysis. CC and LT contributed to starch and secondary metabolite analysis. SD and LT contributed to starch and sugar content analysis. LU helped with interpretation of chlorophyll fluorescence data. LT wrote the first draft of the manuscript, BB and J-CB improved it and all authors revised it.

## Conflict of Interest Statement

The authors declare that the research was conducted in the absence of any commercial or financial relationships that could be construed as a potential conflict of interest.
